# Propionate Induces Virulent Properties of Crohn’s Disease-Associated *Escherichia coli*

**DOI:** 10.3389/fmicb.2020.01460

**Published:** 2020-07-08

**Authors:** Olga V. Pobeguts, Valentina G. Ladygina, Daria V. Evsyutina, Artem V. Eremeev, Aleksandr I. Zubov, Daria S. Matyushkina, Peter L. Scherbakov, Daria V. Rakitina, Gleb Y. Fisunov

**Affiliations:** ^1^Department of Molecular Biology and Genetics, Federal Research and Clinical Centre of Physical and Chemical Medicine, Federal Medical-Biological Agency, Moscow, Russia; ^2^A. S. Loginov Moscow Clinical Scientific Center, Moscow, Russia

**Keywords:** Crohn’s disease, Crohn’s disease-associated adherent-invasive *Escherichia coli*, propionic acid, 2D electrophoresis, macrophages

## Abstract

Crohn’s disease (CD) is a severe chronic immune-mediated granulomatous inflammatory disease of the gastrointestinal tract. The mechanisms of CD pathogenesis remain obscure. Metagenomic analysis of samples from CD patients revealed that several of them have the elevated level of *Escherichia coli* with adhesive–invasive phenotype (AIEC). Previously, we isolated an *E. coli* strain CD isolate ZvL2 from a patient with CD, which features AIEC phenotype. Here, we demonstrate that prolonged growth on propionate containing medium stimulates virulent properties of CD isolate ZvL2, while prolonged growth on glucose reduces these properties to levels indistinguishable from laboratory strain K-12 MG1655. Propionate presence also boosts the ability of CD isolate ZvL2 to penetrate and colonize macrophages. The effect of propionate is reversible, re-passaging of CD isolate on M9 medium supplemented with glucose leads to the loss of its virulent properties. Proteome analysis of CD isolate ZvL2 growth in medium supplemented with propionate or glucose revealed that propionate induces expression porins OmpA and OmpW, transcription factors *PhoP* and *OmpR*, and universal stress protein *UspE*, which were previously found to be important for macrophage colonization by enteropathogenic bacteria.

## Introduction

**FIGURE 1:**
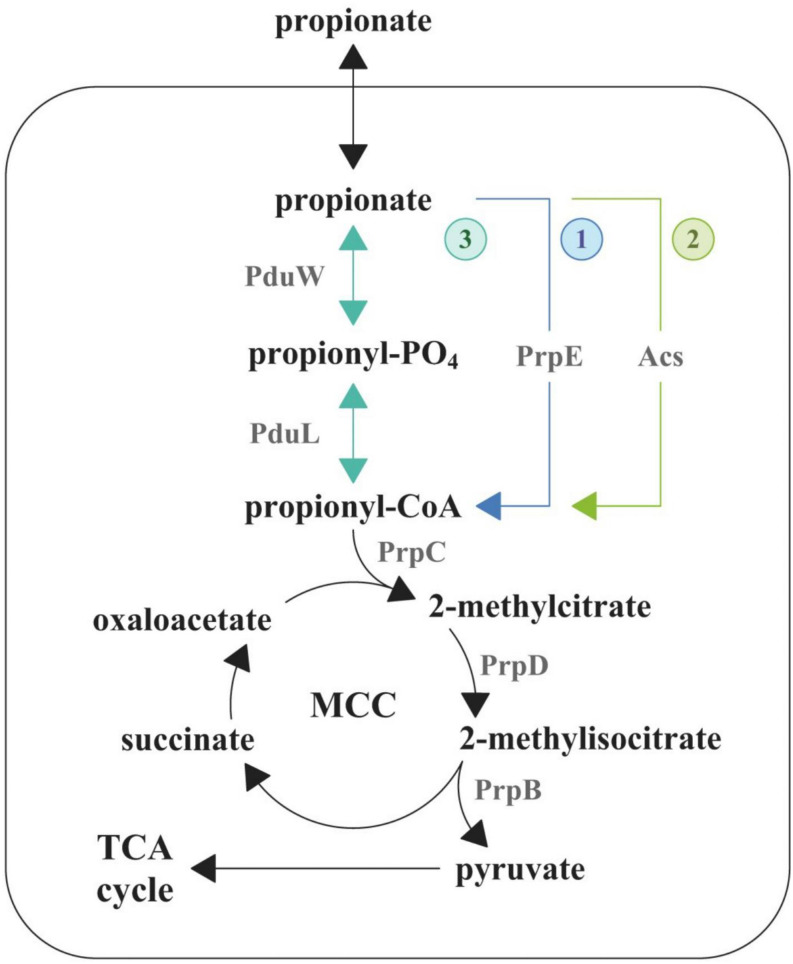
Degradation pathway of propionate in *E. coli*. MCC, methylcitrate cycle; TCA, tricarboxylic acid cycle.

Crohn’s disease (CD) is a severe chronic immune-mediated granulomatous inflammatory disease of the gastrointestinal tract. It can affect each and every region of the gastrointestinal tract from the oral cavity to the rectum. Key signs of the CD include diarrhea, intestinal bleeding, abdominal pain, anemia, and weight loss (Ng et al., 2018). The CD pathogenesis is a multifactorial process, which includes genetic factors, gut microbiota composition, and immune-mediated damage of the intestine (Torres et al., 2017). The mechanisms and the causes of the CD are being extensively studied in recent years but remain obscure to a large extent. The metagenomic analysis of patients’ gut microbiota demonstrated the characteristic increase of representation of *Escherichia coli* strains (Gevers et al., 2014), several of which are able to penetrate the mucin layer, adhere to the epithelial cells, cross the epithelial barrier, and colonize macrophages (Rhodes, 2007). These *E. coli* can modulate macrophages’ defensive functions to avoid lysis (Bringer et al., 2006). These strains were collectively assigned to the adhesive–invasive group of *E. coli* (AIEC). It has been demonstrated that AIEC do not feature any known genetic determinants that are characteristic to enteroinvasive, enteropathogenic, and enterotoxigenic *E. coli* or *Shigella flexneri* (like invasin or intimin of enteropathogenic *E. coli*) (Boudeau et al., 2000). It was shown that the AIEC infection is associated with the release of pro-inflammatory cytokines (Lapaquette et al., 2012). It indicates that colonization of macrophages leads to an increase of inflammation, which in turn increases the amount of new macrophages, which are used for further colonization. AIEC were introduced as a pathotype along with the role of agent provocateur they were supposed to play in the CD pathogenesis (Strober, 2011). Their pathogenic potential was supported by phylogenetic relationship observed between genomes of some AIEC and extracellular pathogenic *E. coli*, which causes urinary tract infections and neonatal meningitis (Miquel et al., 2010; Nash et al., 2010). In addition, Schippa et al. found a significantly higher representation of virulent genes in AIEC from CD patients. These studies have also confirmed the “pathobiont” nature of AIEC strains (Schippa et al., 2012; Conte et al., 2014). In addition, AIEC differ from the opportunistic pathogens because their pathogenic effect is mediated by the stimulation of the host’s immune system (Hornef, 2015). The determinants that are responsible for transition to the adhesive–invasive phenotype and the macrophage colonization are still unknown. In order to outcompete the species of normal gut microbiota, pathogens use different mechanisms including inflammation induction, direct or indirect eradication of commensal species, and use of alternative nutrients (Stecher, 2015). There is a growing amount of data that indicate that the majority of enteropathogens feature specific metabolic pathways, which allow them to use alternative carbon sources (Staib and Fuchs, 2014). It has been demonstrated that they are able to utilize ethanolamine, which is formed during catabolism of phospholipids, fucose or rhamnose, propionate, and several other metabolites that are unavailable for the commensal bacteria (Ferreyra et al., 2014; Staib and Fuchs, 2014; Ormsby et al., 2018, 2019). Due to that fact that the study of metabolic pathways that aid macrophage colonization is an urgent task for researchers, earlier we sequenced 28 *E. coli* isolates from 10 patients with CD (Rakitina et al., 2017). In this work, we aimed to study the effect of different carbon sources including glucose and propionate on the adhesive–invasive phenotype and the ability to survive in macrophages of a selected *E. coli* isolate, CD isolate ZvL2, which is one of the isolates obtained by our group in 2017 (isolate RCE07, Rakitina et al., 2017). Propionic acid is a common gut metabolite. It is known that short-chain fatty acids are naturally formed as the metabolites of the normal gut microbiota in anaerobic conditions. Up to 90% of the fatty acid species within the gut represent acetic, butyric, and propionic acids (Cummings et al., 1987). Of these three species, propionate and acetate can be utilized by *E. coli*. It metabolizes propionate via the 2-methylcitrate cycle (Figure 1) (Ormsby et al., 2019). This pathway is initiated via the activation of propionate to propionyl-CoA by three pathways. The first pathway is common for Enterobacteriales and is catalyzed by propionyl-CoA synthetase (PrpE) (Ormsby et al., 2019). This gene is located in the *prpBCDE* operon with other members of 2-methylcitrate cycle and is regulated by the PrpR transcriptional regulator. These genes form the *prpR prpBCDE* divergon (Suvorova et al., 2012). The second pathway is catalyzed by acetyl-CoA synthetase (Acs), which is under post-translational control by acetylation by CobB (Liu et al., 2014). The third way is a two-step conversion of propionate to propionyl-phosphate by propionate kinase (PduW) and then phosphate propionyl transferase PduL converts propionyl-phosphate to propionyl-CoA. The third pathway has been identified in *Salmonella enterica* serovar Typhimurium (Palacios et al., 2003; Liu et al., 2007). The subsequent condensation of oxaloacetate with propionyl-CoA is catalyzed by the 2-methylcitrate synthase (PrpC) resulting in the production of 2-methylcitrate. In the next step, the 2-methylcitrate dehydratase (PrpD) isomerizes 2-methylcitrate to 2-methylisocitrate, which is further cleaved to pyruvate and succinate by the 2-methylisocitrate lyase (PrpB).

It was shown previously that the propionate can stimulate adhesive–invasive properties and acid stress resistance of AIEC (Ormsby et al., 2018, 2019), unlike antimicrobial effect on *Salmonella typhimurium* (Hung et al., 2013). In addition, Ormsby et al. (2019) identified that the propionate serves as a metabolic signal, which stimulates utilization of ethanolamine. It is still unknown how propionate induces the adhesive–invasive properties of *E. coli* and its ability to survive in macrophages. In order to identify the major changes that occur upon change of carbon source, we carried out comparative proteome analysis of CD isolate of *E. coli* grown on glucose and propionate using 2D electrophoresis.

## Materials and Methods

### Cell Cultures

*Escherichia coli* isolate ZvL2 (CD isolate ZvL2) was obtained by an endoscopic surgery of a patient with CD at The Loginov Moscow Clinical Scientific Center. Sample collection was carried out in accordance with the requirements of the local Ethics Committee based on the informed consent of the patient. CD isolate ZvL2 was sequenced by our group (isolate RCE07, Rakitina et al., 2017) and identified that it belongs to phylogroup A. Laboratory strain K-12 MG1655 and CD isolate ZvL2 were cultivated on M9 medium with a supplement of 50 mM glucose (Glu) or 20 mM sodium propionate (PA) at 37°C and 180 r/min. Growth curve was monitored using OD at 600 nm. R package Growthcurver was used to fit growth curve data to the logistic equation (Sprouffske and Wagner, 2016). The value of arguments (r—the growth rate, n0—the initial population size, t_gen—the maximum doubling time, etc.) was extracted (Supplementary Figure S2 and Supplementary Table S3).

Caco-2 cell line was cultivated in DMEM with the addition of 10% of bovine fetal serum at 37°C and 5% CO_2_. THP-1 cell line was cultivated in RPMI 1640 medium with the addition of 10% of bovine fetal serum at 37°C and 5% CO_2_. Macrophages were differentiated using the standard 72-h protocol using 200 nM 13-phorbol-12-myristate acetate (Starr et al., 2018).

### RNA Purification and cDNA Synthesis

RNA isolation was performed as described above (Gorbachev et al., 2013). One-hundred-microliter aliquots of *E. coli* culture were directly lyzed in TRIzol LS reagent (Life Technologies) at a 1:3 ratio of culture medium:TRIzol LS (v/v). The nucleic acids were extracted with chloroform and precipitated by the addition of an equal volume of isopropanol and subsequent centrifugation. The pallets were washed with 80% ethanol and finally resuspended in 20 μl of mQ (Panreac). The amount of RNA was determined using the Qubit 2.0 fluorometer (Thermo Fisher Scientific). The resulting RNA was treated by DNAse I (Thermo Scientific), and cDNA was synthesized from random hexamer primers by Maxima H Minus Reverse Transcriptase (Thermo Scientific) according to the manufacturer’s protocol.

### qRT-PCR

Quantitative real-time PCR was performed using dNTP, PCR buffer, Taq-polymerase (Lytech), SYBR Green I (Invitrogen), and CFX96^TM^ Real-Time PCR Detection System (Bio-Rad) PCR machine. Primers used are listed in Supplementary Table S1. All primers were designed using BAC-Browser (Garanina et al., 2018). Each 20-μl reaction contained 0.2 μl of template cDNA. Thermal cycling conditions were as follows: initial denaturation at 95°C for 1 min, then 40-cycle amplification (94°C for 15 s, 58°C for 20 s, and 68°C for 1 min) with a single fluorescence per reading. Melting curve was obtained by gradually heating the PCR mixture from 65 to 94°C at a rate of 0.5°C every 5 s, with continuous fluorescence scanning. Relative expression for each sample was determined using the 2^–ΔΔCt^ method with normalization to the amount of *gyrA* transcripts present in the RNA samples. qRT-PCR experiments were carried out on three biological replicates per strain, for each condition. Significance of changes was calculated using *t*-test in R.

The efficiency of the PCR amplification was determined for all primer pairs. Standard curves were plotted for five twofold serial dilutions of cDNA. The slope of the standard curve was used to calculate the PCR efficiency. For all primers, PCR efficiency was 96–104%. Two technical repeats were used (Supplementary Table S2 and Supplementary Figure S1).

### DNA Extraction

*Escherichia coli* cells were harvested by centrifugation (5000 *g* for 5 min, 4°C) and lyzed with CTAB buffer [2% CTAB, 100 mM Tris–HCl (pH 8.0), 20 mM EDTA, and 1.4 M NaCl] at 60°C for 30 min with subsequent chloroform extraction (1:1) and isopropanol precipitation (1:1) with the addition of 10% v/v 3M sodium acetate (pH 5.2) (Stewart and Via, 1993). The amount of DNA was determined using the Qubit 2.0 fluorometer (Thermo Fisher Scientific). The genomic DNA of laboratory strain K-12 MG1655 and CD isolate ZvL2 was used as matrix for PCR amplification of *acs*, *cobB*, *prpE* genes, and intergenic regions of *prpR*, *prpB*, and *prpC.* PCR was performed using dNTP, PCR buffer, Taq-polymerase (Lytech), and Tetrad 2 Peltier Thermal Cycler (Bio-Rad). Primers used are listed in Supplementary Table S1. Thermal cycling conditions were the same as that described in qRT-PCR section with a few differences: only 22 cycles of PCR were used and the duration of elongation step depended on the final amplicon length, assuming that processivity of Taq-polymerase (Lytech) is 1000 bp/s. Agarose gel electrophoresis was performed to verify the size and purity of the PCR products [1.5% w/v agarose, 90 mM Tris-borate (pH 8.3), and 0.5 μg/ml ethidium bromide]. The sequences of the amplicons were obtained by Sanger dideoxy sequencing method using Bigdye Terminator v.3.1 Cycle Sequencing Kit and ABI Prism Genetic Analyzer 3730XL following the manufacturer’s instructions (Applied Biosystem).

### Analysis of Adhesive and Invasive Properties of *E. coli*

Analysis of adhesive–invasive potential was carried out on Caco-2 cells as described earlier (Olivares-Morales et al., 2018), with some modifications. The layer of Caco-2 cells was infected by CD isolate ZvL2 at 1:10 ratio. Prior to infection, *E. coli* cells were diluted by sterile PBS buffer to 0.1 OD_600_ centrifuged at 5000 *g* for 5 min, washed again in PBS, and then centrifuged and resuspended in 1 ml of DMEM. The infected cells were incubated for 3 h at 37°C and 5% CO_2_. After incubation, a subset of the wells was washed two times by PBS to remove non-attached bacteria. Then 0.5 ml of 0.5% Triton X-100 in PBS was added to the wells and incubated for 5 min. The lysate was collected into 0.5 ml of LB broth. These samples contained cells that were attached to or engulfed in Caco-2 cells. The rest of the wells were incubated for 2 h more. After that, the medium was removed and fresh aliquots of 1 ml of DMEM supplied with gentamicin (300 μg/ml) were added and the cells were incubated for 1 h at 37°C and 5% CO_2_. After that, the wells were washed with PBS and the Caco-2 cells were lyzed by Triton X-100 as described above. The obtained samples of *E. coli* in Caco-2 lysates were plated on solid LB agar and cultivated overnight at 37°C. Then, the numbers of CFUs were calculated (Supplementary Figure S3) and the values of percent adhesion–invasion and invasion compared to the initial inoculum were determined. The analysis has been carried out in three biological replicates.

### Analysis of Survival in Macrophages

The survival rate of laboratory strain K-12 MG1655 and CD isolate ZvL2 was assayed as described earlier (Migliore et al., 2018) with some modifications. Macrophages of the THP-1 cell line were infected by *E. coli* in a 1:100 ratio. *E. coli* cells were washed from the medium as described above and resuspended in RPMI 1640 medium. One milliliter of the suspension was added per well. The plates were centrifuged at 1000 *g* for 10 min and incubated for 1 h at 37°C and 5% CO_2_to allow internalization. Cell monolayers were washed twice in PBS to remove extracellular bacteria and treated with RPMI containing gentamicin (300 μg/ml; 20-fold the MIC for both CD isolate ZvL2 and MG1655) for 1 h to kill non-internalized bacteria. Subsequently, monolayers of part of wells were washed twice with sterile PBS and lyzed by the addition of deionized water with 0.5% (vol/vol) Triton X-100 for 5 min, to release internalized bacteria. In order to obtain the bacterial titer precisely, cell lysates were serially diluted, 50 μl of each dilution was plated on solid LB agar and incubated at 37°C overnight, and CFU (and thus the number of internalized bacteria) were determined (1 h postinfection). The remaining unlyzed monolayers were incubated for 6 and 24 h. After incubation, the monolayers were washed twice with sterile PBS and lyzed in deionized water containing 0.5% (vol/vol) Triton X-100 for 5 min to release the bacteria that have survived the incubation. Dilution series of the lysates were plated on solid LB agar and incubated at 37°C overnight. Then, the numbers of CFUs were calculated (Supplementary Figure S4) and the number of bacterial populations that survived during 6 and 24 h incubation in macrophages was estimated (6 h and 24 h postinfection). The analysis has been carried out in six biological replicates.

### 2D-DIGE of CHAPS-Soluble Fraction of *E. coli*, Tryptic Digestion, and Protein Identification

Differential 2D gel-electrophoresis, tryptic digestion of the proteins, and protein identification by MALDI-ToF mass spectrometry were performed as described above (Fisunov et al., 2011). The gels were scanned on a Typhoon Trio (Amersham) scanner at 532 nm (Cy3) and 633 nm (Cy5) 500 pmt laser intensity. Quantitative analysis was performed using PDQuest 8.0 software (Bio-Rad). For spot excision, the gels were stained by silver as described by Shevchenko et al. (1996). The spots of interest were excised and washed in mixing solution containing 15 mM tetrathionate and 50 mM potassium ferrocyanide. Then, the gel pieces were washed in mQ water until the yellow color disappeared. Then, gel pieces were dried in 100% acetonitrile. Three to four microliters of trypsin solution (40 mM ammonium bicarbonate, 10% acetonitrile, and 40 nM trypsin) was added to each sample and the samples were incubated for 30 min on ice and subsequently for 16–17 h at 37°C. Peptide extraction was performed by addition of 0.5% v/v TFA in mQ water. The samples were incubated in an ultrasonic bath for 10 min and then incubated for 1 h at room temperature. Mass spectrometric analysis was performed on Ultraflex II MALDI-ToF-ToF (Bruker Daltonics) as described earlier (Zgoda et al., 2006). Proteins were identified against the protein database of CD isolate ZvL2 (Rakitina et al., 2017). The identification cutoff was 44 (*p* < 0.05).

### Statistical Analysis

Descriptive statistics of mean values, standard deviation (SD), and confidence intervals (CI) were performed using Origin 8.2 software (OriginLab, Northampton, MA, United States). Spearman rank correlation was calculated using R-3.6.3.

## Results

### Comparison of Genes and Pathways Related to Propionate Degradation in CD Isolate ZvL2 and *E. coli* K12-MG1655

**FIGURE 2:**
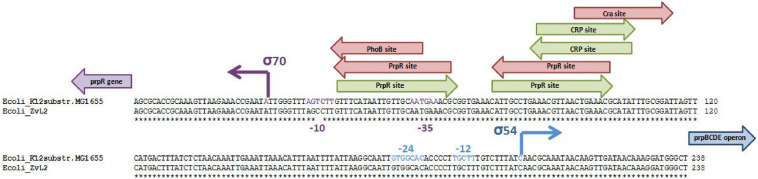
Comparison of intergenic region of *prpR prpBCDE* divergon of ZvL2 and K12-MG1655 strains. Arrows indicate transcription factor binding sites, red for repressor and green for activator.

**FIGURE 3:**
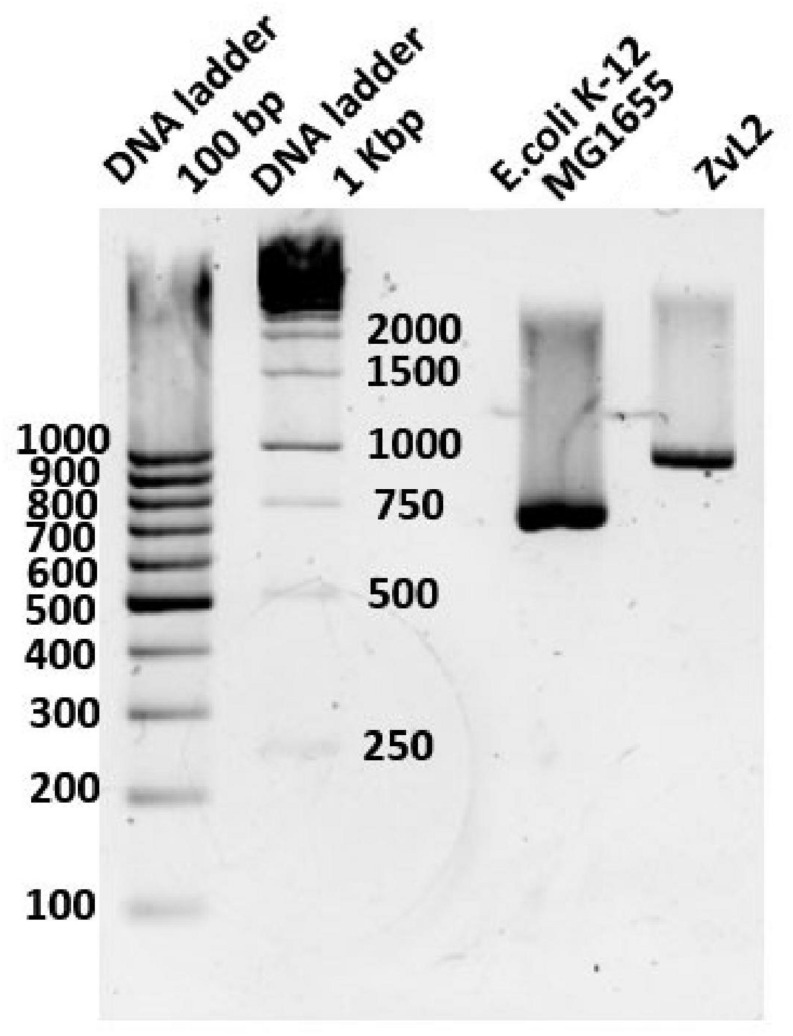
The difference of intergenic length between *prpB* and *prpC* genes in *E. coli* K12-MG1655 and ZvL2.

We compared sequences of genes involved in metabolism of propionate in isolates ZvL2 and *E. coli* K12 MG1655. Some genes (*acs*, *cobB*, and *prpE*) have frameshifts in the genome of ZvL2 probably due to the sequencing error. We amplified these genes from genomic DNA with specific primers and sequenced using the Sanger method. There were no frameshifts found in these genes. The comparison of the intergenic regions of *prpR* and *prpBCDE* of ZvL2 and K-12 MG1655 revealed several differences: first, substitution in -10-box of *prpR*, the activator of *prpBCDE* (Figure 2), and second, there are different amounts of repeats between *prpB* and *prpC.* These genes are transcribed as polycistronic mRNA under aerobic conditions. However, under anaerobic conditions, the transcription of each gene is regulated separately. It has been proposed that the regulation takes place on a post-transcriptional level. The key role in this process is played by RNase R, which may target the repeats between these genes (Simonte et al., 2017). The difference in the number of repeats between *prpB* and *prpC* was confirmed by PCR and Sanger sequencing (Figure 3). The comparison of the coding sequences of *prpBCDE* did not reveal any differences. The coding sequence of *prpR* features few amino acid substitutions (SAPs), but they do not seem to impact its function. Search for *pduW* and *pduL* homologs in K-12 MG1655 and ZvL2 revealed that both encode protein TdcD that share 44% sequence identity with PduW of *Salmonella* and 99% between each other. Substitutions in *tdcD* of K-12 MG1655 and ZvL2 are not located in critical positions. *pduL* homolog was found only in ZvL2 with 80% of sequence identity. Alternatively, the function of *pduL* may be taken by *pta* (phosphate acetyltransferase), which is common for both strains (Palacios et al., 2003). Thus, the main differences between ZvL2 and K-12 MG1655 are mutation in the -10 box of *prpR*, different amount of repeats between *prpB* and *prpC*, and absence of *pduL* in the laboratory strain.

### CD Isolate ZvL2 Shows Faster Adaptation to M9 Minimal Medium Supplied With Propionate

**FIGURE 4:**
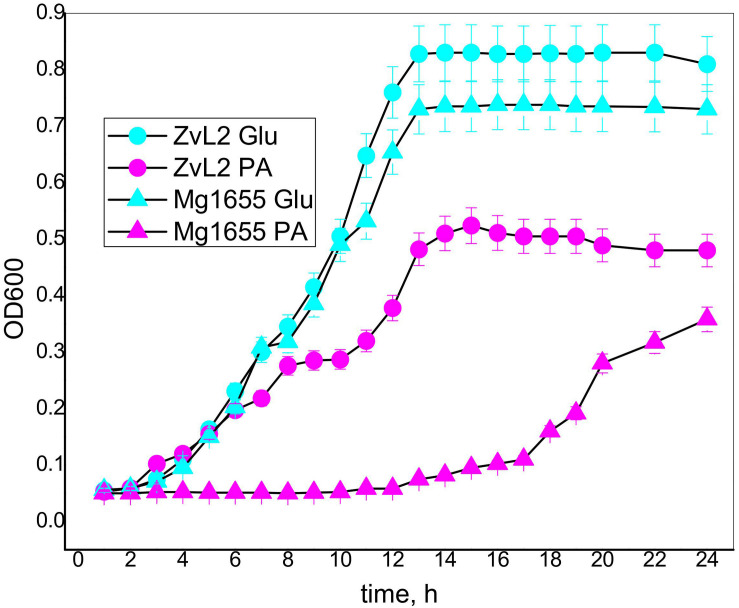
The growth curves of the CD isolate ZvL2 and laboratory strain K 12Mg1655 in minimal media M9 supplemented with glucose (ZvL2Glu) (50 mM) or sodium propionate (ZvL2PA) (20 mM) as a carbon source.

Crohn’s disease isolate ZvL2 and K-12 MG1655 laboratory strain were cultivated on M9 minimal medium with the addition of either 50 mM of glucose (Glu) or 20 mM of sodium propionate (PA). The amount of supplied propionate was chosen taking into account the data on concentration of propionic acid in the human gut. It was demonstrated that its concentration varies across the different parts of the gastrointestinal tract and reaches a maximum of 27 mM in the colon (Ormsby et al., 2018). Figure 4 demonstrates growth curves of CD isolate ZvL2 and K-12 MG1655 laboratory strain on M9 minimal medium in the presence of glucose and propionate. The growth rates of both strains on glucose medium are similar. However, growth curves on propionate show drastic differences. CD isolate ZvL2 grows significantly faster than K-12 MG1655. The growth rate of CD isolate ZvL2 on propionate at the first 6 h does not differ from the growth rate on glucose, while K-12 MG1655 on propionate showed almost no signs of growth at all. The growth rate of K-12 MG1655 on propionate can be evaluated only about 12 h later than CD isolate ZvL2.

To further assess the growth parameters of the strains, we modeled the experimental data using the non-linear regression with Verhulst equation. The model predicted doubling time and maximum population capacity (Crow and Kimura, 1970; Rockwood, 2015; Sprouffske and Wagner, 2016). The population capacity was comparable for each of the strains on the same energy source and was higher on glucose than on PA. The doubling time was not significantly different for both strains under both conditions (Supplementary Figure S2 and Supplementary Table S3). The doubling time of K-12 MG1655 was about 1.3 h at both conditions. For ZvL2, it was about 1.3 h on glucose, but 1.8 h on PA. This indicates that the retarded growth of K-12 MG1655 is due to the longer lag phase. One can speculate that K-12 MG1655 cells die on PA, and the released nutrients are used by survivors for further growth. However, only 5% (v/v) of cell culture is transferred in the fresh medium for the experiment. Thus, the amount of spare biomass provided by dead cells is negligible compared to final biomass, so it cannot reach the observed values without the use of PA from the medium. We propose that K-12 MG1655 strain needs time to adapt to growth on PA, while ZvL2 is prepared for it. Later, the growth rate of CD isolate ZvL2 on propionate decreases, probably due to inhibition of fructose-1,6-bisphosphatase by increasing concentration of 2-methylisocitrate (Rocco and Escalante-Semerena, 2010).

### Propionate Induces Expression of Operon Only in ZvL2 Isolate

**FIGURE 5:**
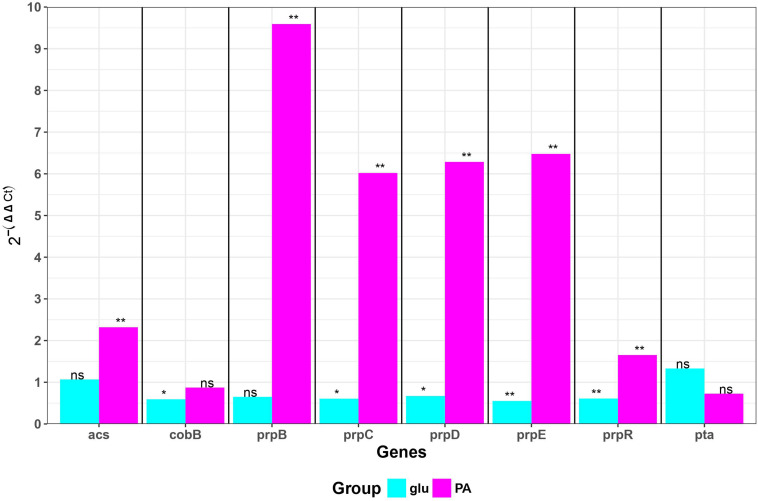
Differential gene expression of *E. coli* K-12 MG1655 and ZvL2 grown in minimal M9 medium supplemented either 50 mM of glucose (glu) or 20 mM of sodium propionate (PA). Transcript levels in the ZvL2 isolate are presented as a fold difference compared to those for the *E. coli* K-12 MG1655. Statistical significance between ZvL2 and *E. coli* K-12 MG1655 was determined by Student’s *t-*test (*P ≤ 0.05, **P ≤ 0.01, ns, non-significant).

We measured transcription of genes involved in propionate utilization in K-12 MG1655 and ZvL2 strains in M9 medium supplied with glucose and propionate using RT-qPCR (Figure 5). Transcription of *prpBCDE* operon was significantly induced only by propionate in ZvL2 strain compared to K-12 MG1655. In particular, induction of *prpB* was higher than that of the downstream genes of the operon. In addition, propionate induced transcription of acetyl-CoA synthase (*acs*) in ZvL2 strain. The ability to upregulate expression of propionate utilization enzymes may explain the observed difference in adaptation of ZvL2 and K-12 MG1655 to propionate.

### Propionic Acid Exacerbates and Glucose Reduces the Adhesive and Invasive Phenotype of AIEC

**FIGURE 6:**
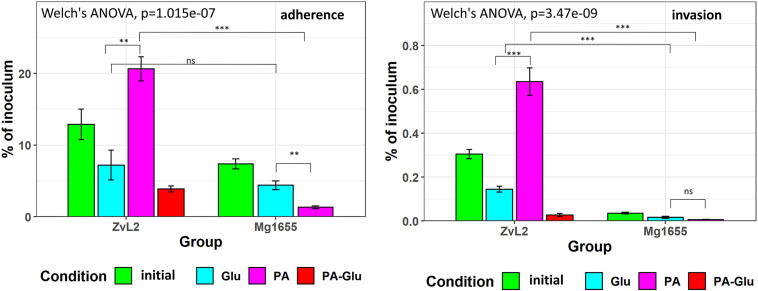
Adhesion and invasion of CDprotect isolate ZvL2 and K-12 MG1655 laboratory strain to intestinal epithelial cells Caco-2. ZvL2, Mg1655—after one passage and ZvL2 Glu, Mg1655 Glu—after five passages from colony on minimal M9 medium supplemented with glucose, ZvL2 PA, Mg1655 PA—after five passages from colony in minimal M9 medium supplemented with sodium propionate. ZvL2 PA-Glu—re-passaging on M9 medium supplemented with glucose after sodium propionate. The kinetics of adhesion and invasion were measured up to 3 and 5 h at an MOI of 10. Adhesion and invasion were also represented as a percentage of initial inoculums. Data are expressed as the means ± SD from three independent experiments. *P ≤ 0.05, **P ≤ 0.01, ***P ≤ 0.001.

We used colon carcinoma cell line Caco-2 to study the adhesive and invasive properties of CD isolate ZvL2 grown on M9 medium supplied with glucose or propionate. We prepared CD isolate ZvL2 by three methods of cultivation: cells grown on M9 medium with glucose for one passage from colony (ZvL2), cells grown on M9 medium with glucose for five passages (ZvL2Glu), and cells grown on M9 medium with propionate for five passages (ZvL2PA). The control laboratory strain was treated in the same fashion. Figure 6 demonstrates that cultures ZvL2 and ZvL2PA feature adhesive–invasive phenotype. The rate of adhesion–invasion is 15% and 20% and the rate of invasion is 0.3 and 0.64% for ZvL2 and ZvL2PA, respectively, though passaging on glucose (ZvL2Glu) reduces adhesion–invasion rate to 7% and invasion rate to 0.14%. Passaging on both glucose and propionate of K-12 MG1655 did not increase adhesive and invasive properties. Thus, long-term growth on glucose drastically reduces the adhesive and invasive properties of CD isolate ZvL2, while propionate induces adhesive–invasive phenotype. However, we did not observe the effect of propionate on K-12 MG1655.

### Propionate Increases and Glucose Decreases the Survival Rate of CD Isolate ZvL2 in Macrophages

**FIGURE 7:**
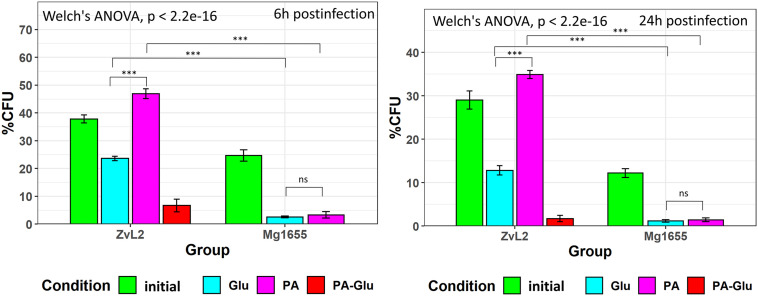
Survival and replication in macrophages (THP-1 cell line) of CD isolate ZvL2 and laboratory strain K12 Mg1655 grown on minimal M9 medium supplemented with glucose (Glu) or sodium propionate (PA) [ZvL2, K12 Mg1655—after one passage from colony on minimal M9 medium supplemented with glucose, ZvL2 Glu, Mg1655 Glu—after five passages from colony on minimal M9 medium supplemented with glucose, ZvL2 PA, Mg1655 PA—after five passages from colony in minimal M9 medium supplemented with sodium propionate after 6 (6 h) and 24 h (24 h) postinfection]. ZvL2 PA-Glu—re-passaging on M9 medium supplemented with glucose after sodium propionate. 1hPI, the number of internalized bacteria during 1 h of postinfection. The number of internalized bacteria was, on average, three *E. coli* per macrophage. For all values, the mean ± SD of six independent biological replicates are shown. *P ≤ 0.05, **P ≤ 0.01, ***P ≤ 0.001.

Since macrophage infection is an important component of CD pathogenesis, we tested the ability of CD isolate ZvL2 to colonize macrophages. The efficiency of phagocytosis of CD isolate ZvL2 and K-12 MG1655 strain by human macrophages and the survival rate of the strains were measured by gentamicin test using THP-1 macrophage cell line. Macrophages were cultivated for 1 h with the *E. coli* strains. Then, the macrophages were treated with gentamicin to remove *E. coli* that were not internalized by macrophages. The part of wells was washed twice with sterile PBS and lyzed by the addition of deionized water with 0.5% (vol/vol) Triton X-100 for 5 min, to release internalized bacteria. In Figure 7, at 1 h PI is the number of internalized bacteria during 1 h of postinfection. The average number of internalized bacteria was three *E. coli* per macrophage. The survival rate was assayed as percent of the remaining CFU after 6 and 24 h of cultivation. We demonstrate that CD isolate ZvL2 is more resistant to macrophages’ lysosomes compared to K-12 MG1655 strain. The survival rate of CD isolate ZvL2 is 41 and 28% after 6 and 24 h, respectively. In comparison, the survival rate of K-12 MG1655 is 20.5 and 11.7%. The ability of CD isolate ZvL2 to survive in macrophages decreases to the values of laboratory strain after the passaging on glucose. In particular, 6 and 24-h survival rates decreased to 24 and 10.6%, respectively. In contrast, passaging on propionate increases survival rate to 49 and 35%, respectively. We did not observe the effect of propionate for K12 MG1655 (after five passages on this carbon source) after 6 and 24 h of postinfection. The effect of propionate is reversible, re-passaging of CD isolate on M9 medium supplemented with glucose after five passages on propionate leads to the drastic decrease of the survival rate.

Thus, we obtained two states of CD isolate ZvL2—active, which is characterized by adhesive–invasive phenotype and resistance to macrophages, and inactive, which is similar to laboratory strain in terms of adhesion, invasion, and survival in macrophages. Active state is induced and supported by propionate but deteriorates by growth on glucose.

### The Proteome of CD Isolate ZvL2 Undergoes Rearrangement During Growth on Glucose or Propionate

**FIGURE 8:**
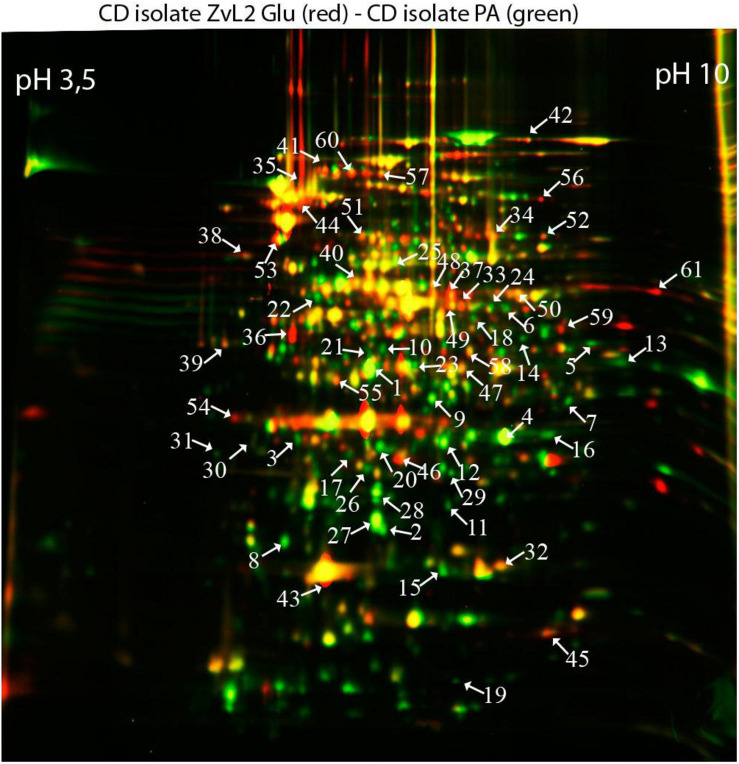
Comparative proteomic analysis of CD isolate ZvL2 grown on glucose (Glu) and propionate (PA) for five passages revealed by differential 2D gel electrophoresis (pH range 3.5–10). Red corresponds to cells grown on glucose, while green corresponds to cells grown on propionate. The cutoff score for protein identification in the mascot engine was 44 (*p* < 0.05). The numbers of protein spots indicated by the arrows correspond to the numbers in Tables 1, 2.

**FIGURE 9:**
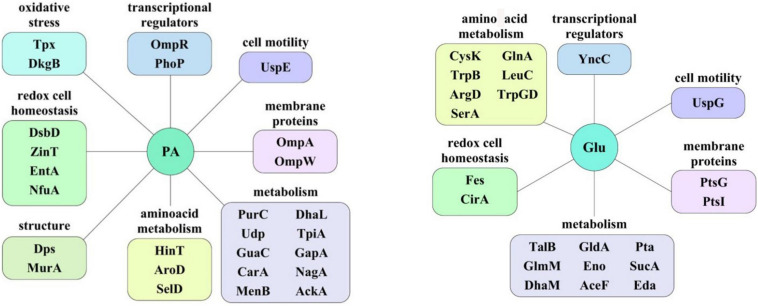
The protein groups whose representation increases depending on the carbon source. PA, sodium propionate; Glu, glucose.

In order to reveal the physical basis of the observed phenotypic changes of CD isolate ZvL2 grown on different substrates, we performed proteome analysis using 2D differential electrophoresis. Figure 8 shows 2D differential proteome map of CD isolate ZvL2 grown on glucose and propionate for five passages. Red corresponds to cells grown on glucose, while green corresponds to cells grown on propionate. The cutoff score for protein identification in the mascot engine was 44 (*p* < 0.05). A protein was identified as significantly changed if the fold change was greater than 2 (up and down). Finally, we identified 60 differentially produced proteins (Tables 1, 2). First, we have to note the upregulation of porins *OmpA* and *OmpW* during growth on propionate. *OmpA* was previously identified as a pathogenicity factor that mediates colonization of macrophages by pathogenic *E. coli* (Sukumaran et al., 2003). Further significant amount of upregulated proteins is involved in RedOx homeostasis or protection from reactive oxygen species (Figures 8, 9). They include thioredoxin peroxidase *BtuE*, thiol peroxidase *Tpx*, thiol:disulfide interchange protein *DsbA*, cysteine desulfurase *IscS*, Fe/S-clusters biogenesis protein *NfuA*, NAD(+) synthetase *NadE*, menaquinone biosynthesis protein *MenB*, and selenophosphate synthase *SelD*. In addition, there are two upregulated proteins that are involved in DNA protection: *UspE* and *Dps*. The latter also functions as iron storage and protects from oxidative stress caused by free Fe^2+^. Upregulation of *UspE* during growth on propionate is coupled with downregulation of its antagonist *UspG*. Another abundant group of upregulated proteins is linked to cell wall biogenesis: UDP-*N*-acetylglucosamine 1-carboxyvinyltransferase *MurA*, *N*-acetylglucosamine-6-phosphate deacetylase *NagA*, and endolytic peptidoglycan transglycosylase *RlpA*. Another interesting protein identified as significantly changed in our study is *Slp*. This protein has been shown to bind *PIgR* receptor on gut epithelium by enterohemorragic *E. coli* (Fedorchuk et al., 2019) and to play an important role in adherence. However, we identified decrease rather than increase of *Slp* during growth on propionate (Figure 9). Among the differentially expressed proteins identified by 2D electrophoresis, we found three transcription factors: *OmpR*, *PhoP*, and *McbR*. These proteins are master regulators. *PhoP* is a subunit of a two-component system with *PhoQ*. It is involved in the adaptation to low Mg^2+^ concentrations and oxidative stress (Kato et al., 1999). Recently, it has been shown that the *PhoP/PhoQ* signal pathway is involved in extraintestinal pathogenic *E. coli* survival in macrophages (Zhuge et al., 2018). *OmpR* is a global regulator that controls oxidative and osmotic stress and is involved in the regulation of virulence and metabolism (Chakraborty and Kenney, 2018). *McbR* increases biofilm formation by repressing overproduction of the exopolysaccharide identified as colanic acid (Zhang et al., 2008). Using RegulonDB database^1^, we searched the targets of the identified regulators within the proteins differentially expressed on 2D EF. We identified only two direct targets: *argD* (acetylornithine/succinyl-diaminopimelate aminotransferase) and *nagA* (*N*-acetylglucosamine-6-phosphate deacetylase). The observed changes may be the result of indirect regulation. For example, *PhoP* regulates eight transcription factors and two ncRNAs (according to RegulonDB).

**TABLE 1:** Proteins upregulated in ZvL2 isolate grown on M9 medium containing PA compared with cultivation on Glu.

	Gene	Protein	Score	ZvL2 PA/ZvL2 Glu
**Membrane protein**				
1	KZO87574.1	Porin *OmpA*	87	4.090.53
2	KZO85236.1	Porin *OmpW*	44	2.750.30
**Nucleotide metabolism**				
3	KZO86959.1	Phosphoribosylaminoimidazole-succinocarboxamide synthase *PurC*	158	2.240.39
4	KZO81989.1	Uridine phosphorylase *Udp*	122	5.800.06
5	KZO86279.1	Guanosine 5’-monophosphate oxidoreductase *GuaC*	71	2.710.30
6	KZO86225.1	Carbamoyl phosphate synthase small subunit *CarA*	77	2.380.28
**Anaerobic growth**				
7	KZO86714.1	Dihydroxynaphthoic acid synthetase *MenB*	84	3.080.40
**Oxidative stress protection**				
8	KZO85295.1	Peroxidase *Tpx*	80	2.950.32
9	KZO82481.1	2,5-diketo-D-gluconicacid reductase *DkgB*	88	3.180.35
**Cell motility**				
10	KZO85303.1	Universal stress protein *UspE*	258	2.350.28
**Energy metabolism**				
11	KZO83151.1	Dihydroxyacetone kinase *DhaL*	136	2.330.28
12	KZO85449.1	Triosephosphate isomerase *TpiA*	177	4.180.54
13	KZO87259.1	Glyceraldehyde-3-phosphate dehydrogenase *GapA*	168	2.370.25
14	KZO81926.1	*N*-acetylglucosamine-6-phosphate deacetylase *NagA*	67	2.010.22
**Chromosome structure**				
15	KZO86055.1	Nucleoid-associated protein *Dps*	147	3.190.35
**Transcription factors**				
16	KZO86488.1	Transcriptional regulator *OmpR*	62	2.980.29
17	KZO84903.1	Transcriptional regulator *PhoP*	131	2.050.24
**Use of alternative carbon sources**				
18	KZO86735.1	Acetate kinase *AckA*	161	2.560.33
**Amino acid biosynthesis and catabolism**				
19	KZO84886.1	Purine nucleoside phosphoramidase *HinT*	47	2.000.11
20	KZO84529.1	3-dehydroquinate dehydratase *AroD*	100	3.670.46
21	KZO87274.1	Selenophosphate synthetase *SelD*	70	2.070.25
**Cell wall synthesis**				
22	KZO87791.1	UDP-*N*-acetylglucosamine 1-carboxyvinyl transferase *MurA*	46	2.190.28
**Other functions**				
23	KZO87292.1	NAD synthetase *NadE*	187	3.870.50
24	KZO87002.1	Cysteine desulfurase *IscS*	61	1.980.15
25	KZO81874.1	Aminoacyl-histidine dipeptidase *PepD*	184	2.540.33
**Proteins with unknown function**				
26	KZO87561.1	Hypothetical protein TH54_02655	92	1.990.13
**Chaperones**				
27	KZO82051.1	Heat shock protein *IbpA*	109	4.010.52
**Redox cell homeostasis**				
28	KZO85494.1	Protein disulfide isomerase *DsbD*	73	3.150.40
29	KZO82128.1	Zinc/cadmium-binding protein *ZinT*	136	1.980.11
30	KZO83979.1	2,3-dihydroxybenzoate-2,3-dehydrogenase *EntA*	66	1.970.11
31	KZO86481.1	Fe/S biogenesis protein *NfuA*	86	1.990.12

**TABLE 2:** Proteins downregulated in ZvL2 isolate grown on M9 medium containing PA compared with cultivation on Glu.

	Gene	Protein	Score	ZvL2 PA/ZvL2 Glu
**Membrane protein**				
32	KZO82406.1	Membrane Protein *Slp* Family	49	0.480.06
33	KZO85423.1	Subunit of phosphoenolpyruate-dependent phosphotransferase sugar transport system EIIB *PtsG*	47	0.370.04
34	KZO83457.1	ABC transport protein	115	0.500.06
35	KZO82314.1	Phosphoenolpyruvate-protein phosphotransferase *PtsI*	392	0.110.01
**Energy metabolism**				
36	KZO82659.1	Transaldolase *TalB*	154	0.130.02
37	KZO87802.1	Phosphoglucosamine mutase *GlmM*	230	0.190.02
38	KZO83152.1	Dihydroxyacetone kinase *DhaM*	180	0.540.02
39	KZO85424.1	Glycerol dehydrogenase *GldA*	58	0.510.02
40	KZO85054.1	Enolase *Eno*	123	0.490.02
41	KZO86288.1	Dihydrolipoyllysine-residue acetyltransferase component of pyruvate dehydrogenase complex *AceF*	79	0.280.02
42	KZO86736.1	Phosphate acetyltransferase *Pta*	217	0.330.04
43	KZO82737.1	2-Oxoglutarate dehydrogenase *SucA*	225	0.440.06
**Elongation factors**				
44	KZO87805.1	Transcription termination protein *NusA*	264	0.220.03
55	KZO83328.1	Elongation factor *Ts* (*EF-Ts*)	48	0.430.02
**Cellmotility**				
45	KZO83969.1	Universal stress protein *UspG*	144	0.540.07
**Use of alternative carbon sources**				
46	KZO87201.1	Keto-deoxy-phosphogluconate aldolase *Eda*	49	0.330.03
**Amino acid biosynthesis**				
47	KZO82316.1	Cysteine synthase *CysK*	225	0.560,07
48	KZO85241.1	Tryptophan synthase beta chain *TrpB*	103	0.350.04
49	KZO86522.1	Acetylornithine aminotransferase *ArgD*	67	0.230.03
50	KZO88029.1	D-3-phosphoglycerate dehydrogenase *SerA*	186	0.440.06
51	KZO85486.1	Glutamine synthetase *GlnA*	171	0.450.06
52	KZO86254.1	Isopropyl malate isomerase *LeuC*	129	0.380.05
53	KZO85242.1	Glutamine amidotransferase *TrpGD*	181	0.210.03
**Other functions**				
54	KZO87777.1	Isoprenoid biosynthesis protein *ElbB*	75	0.480.06
56	KZO81630.1	AMP nucleosidase *Amn*	137	0.450.06
57	KZO87809.1	Polyribonucleotide nucleotidyltransferase *Pnp*	271	0.230.03
58	KZO83340.1	Acetyl-CoA carboxylase carboxyl transferase subunit alpha *AccA*	91	0.440.06
**Redox cell homeostasis**				
59	KZO83987.1	Enterobactin esterase *Fes*	206	0.410.05
60	KZO83732.1	Catecholate siderophore receptor *CirA*	358	0.230.03

## Discussion

We demonstrated that CD isolate ZvL2 features adhesive–invasive phenotype and can efficiently survive in macrophages in comparison to the laboratory strain K-12 MG1655. CD isolate ZvL2 is capable of utilization of propionate, which serves as an inductor of adhesive–invasive and macrophage resistance properties. Similar observations were made for another CD isolate LF82 (Ormsby et al., 2018, 2019). The adhesive–invasive phenotype was found to be dependent on the carbon source. Prolonged growth on glucose reduces pathogenicity to the levels of laboratory strain, while prolonged growth on propionate promotes it. This finding is striking since propionate has been considered as a health-promoting metabolite for the gut (Hosseini et al., 2011). It should be noted that propionate does not have a similar effect on the laboratory strain K12MG1655, despite the fact that it can also adapt and grow on this carbon source. The effect of propionate is reversible, re-passaging of CD isolate on M9 medium supplemented with glucose leads to the loss of its virulent properties. Comparative proteome analysis identified a set of proteins that are potentially important for the observed phenotype switching. They represent porins; transcription factors *OmpR*, *PhoP*, and *McbR*; proteins involved in protection from reactive oxygen species; and proteins involved in cell wall biogenesis (Figure 9).

Porin *OmpA* represents a multi-functional protein that is important for pathogenesis. *OmpA* can block activation of complement via binding to C4-binding protein (Prasadarao et al., 2002). *OmpA* mediates *E. coli* phagocytosis, survival, and growth in macrophages, which in turn may lead to macrophage lysis. The mechanism of *OmpA*-dependent *E. coli* engulfment differs from the *OmpA*-knockout cells. *OmpA*-expressing *E. coli* induce actin condensation in the site of entry and the engulfment does not depend on macrophage integrins (Sukumaran et al., 2003). *OmpW* has been demonstrated to suppress *E. coli* phagocytosis by macrophages (Wu et al., 2013). Thus, we propose that *OmpA* and *OmpW* may work together: *OmpW* blocks normal phagocytosis, while *OmpA* induces abnormal phagocytosis, which instead of bacteria lysis in the phagosome results in their propagation. In addition, it was shown that in AIEC strain LF82, deletion of the *ompA* gene significantly decreased the ability of the bacteria to invade Intestine-407 epithelial cells compared to a wild-type strain and results in a loss of fusion of outer membrane vesicles with the host cell membrane (Rolhion et al., 2010).

Multiple defensive and functionally related proteins are induced after growth on propionate (Figure 9). Peroxidases and proteins involved in Fe–S cluster biosynthesis have a direct role in resistance to oxidative stress. Stress protein *UspE* has multiple roles in adaptation to environmental perturbations (Tkaczuk et al., 2013). It may protect cells from heavy metals (Xu et al., 2016) and regulate the formation of flagella (Hosseini et al., 2011). *UspE* changes expression cooperatively with its antagonist *UspG* (Nachin et al., 2005; Siegele, 2005). Increase of *UspE* occurs simultaneously with decrease of *UspG* and vice versa. *Dps* protein upregulated during growth on propionate serves as DNA protector and in addition stores iron (Kim et al., 2004; Karas et al., 2015). In addition, growth on propionate induces *DhbA*, enzyme involved in enterobactin siderophore synthesis and decreases enterobactin esterase, involved in its degradation. The latter may be important since depletion of iron is an important defensive strategy of hosts against pathogens (Saha et al., 2019). Upregulated during growth on propionate inner membrane protein *DsbD* is a part of the protein complex responsible for stabilization of intramolecular disulfide bridges between cysteine residues of the exported proteins. It is maintained in the reduced form by the periplasmic oxidoreductase *DsbA*. It is known that *DsbA* was required for AIEC strain LF82 to adhere to intestinal epithelial cells and to survive within macrophages (Bringer et al., 2007). The LF82-dsbA mutant did not express flagella and, probably as a consequence of this, did not express type 1 pili. There is evidence that the *dsbA* gene is needed for LF82 bacteria to grow and survive in an acidic and nutrient-poor medium that partly mimics the harsh environment of the phagocytic vacuole. Under such stress conditions, *dsbA* transcription is highly upregulated.

Thus, propionate induces complex reprogramming of pathogenic *E. coli* strain to the process of infection. We found that propionate induced an increased level of proteins, which are known pathogenicity factors, among them, proteins *OmpA*, *OmpW*, *PhoP*, *OmpR*, and *UspE*. The data obtained in this study suggest that *E. coli* gets ready to suppress normal phagocytosis and induce macrophage infection and raises defense against oxidative burst beforehand.

## Conclusion

This study revealed that the activity of *E. coli* with an adhesive–invasive phenotype depends on the carbon source in the culture medium. In particular, the prolonged growth of *E. coli* isolate obtained by our group from a patient with CD on a M9 medium supplemented with sodium propionate significantly stimulates its adhesive–invasive properties and survival in macrophages, while prolonged passaging on a glucose containing medium, on the contrary, significantly reduces these properties. The cultivation of the CD isolate on different carbon sources made it possible to obtain the same isolate in two different states of activity. A comparative proteomic analysis of these states showed that the propionate induces expression of important pathogenic factors, among them porins, transcription factors, proteins involved in the protection against oxidative stress, and cell wall biogenesis.

## Data Availability Statement

The datasets generated and analysed during the current study are partially available in the Supplementary Material. The rest of the data is available from the corresponding author on reasonable request.

## Author Contributions

All authors have made direct experimental and/or intellectual contribution to the work, and have read and approved the final version. OP and VL designed the study, analysis of adhesive and invasive properties and survival in macrophages of *E. coli*, and 2D electrophoresis. AE contributed to cultivation of the cell cultures. DE and GF contributed to quantitative real-time PCR and comparison of genes and pathways related to propionate degradation. DM contributed to mass spectrometric analysis. AZ contributed to text editing and data processing.

## Conflict of Interest

The authors declare that the research was conducted in the absence of any commercial or financial relationships that could be construed as a potential conflict of interest.

## Footnotes

1 http://regulondb.ccg.unam.mx/
