# Supplementary material for: Propionate Induces Virulent Properties of Crohn’s Disease-Associated Escherichia coli
Source: Front Microbiol. 2020 Jul 8;11:1460. doi: 10.3389/fmicb.2020.01460 (PMC7360682; doi:10.3389/fmicb.2020.01460)
Supplement: Supplementary file 1 [file Data_Sheet_1.pdf]

Table S1. List of primers

Name	Sequence
Primers for qRT-PCR	
prpB_F	GCGCCATGATCACAAAATC
prpB_R	GAAATCGATGATTAAAGCCGGTG
prpC_F	CATTGCGAATTTGAAGAAGTGG
prpC_R	GTTTGTCGGCAATATCCCGCG
prpD_F	AAATGTGCTGTTCAAAATCTCC
prpD_R	TAATCTGCCGCCGTTAAGC
prpE_F	GATGTATGGCTATAACGTGCAG
prpE_R	GAGATACTCTCTTCAATCTCGC
prpR_F	CGGCTTCTTCTGCCAGATC
prpR_R	GATGTGTTACAGGCACTGGC
acs_F	CATGTGGTGGTACTGAAGCGTAC
acs_R	CTTCAAACATCAGCGTGGTCGC
cobB_F	AGAAATTCAGCCTAACGCCG
cobB_R	ATCCATGCCGAGTGGCATTTC
pta_F	CGGTTGATGAACAGGGTCGT
pta_R	CTTCGTTGATGATGGTCGCAT
gyrA_F *(reference gene)*	TGGTGACGTAATCGGTAAATACCA
gyrA_R *(reference gene)*	GTTATCAACGAAATCGACCGTC
Primers for PCR and Sanger sequencing	
acs_check_F	TTGTGGGTTACGATGGCATCG
acs_check_R	CTACATTTAACGCTTATGCCAC
cobB_check_F	AACAGAGGTTGCTATGCTGTC
cobB_check_R	TGATAAGCGTAGTGCATCAGG
prpE_check_F	GTAAGGCGTAAGTTCAACAGG
prpE_check_R	GCATCCGACAATAACCTACTC
prpR_check_F	GGATAAAGCGTTCGCGCTGC
prpR_check_R	GCGTTTCAGTTAACGTTTCAGGC
prpC_check_F	CCGGAACGCCAGAAAGTGC
prpC_check_R	GAAATCGATGATTAAAGCCGGTG

Table S2 Cq values for qRT-PCR efficiency test

Cq_rep1	Cq_rep2	Mean_Cq	SD	Dilution	Log2Dilution	Efficiency(%)
acs						
22.71	22.67	22.69	0.03	1	0	97.88
23.95	23.74	23.84	0.15	2	-1	
25.03	24.81	24.92	0.15	4	-2	
25.96	26.02	25.99	0.04	8	-3	
26.77	26.79	26.78	0.02	16	-4	
cobB						
22.45	22.47	22.46	0.01	1	0	101.16
23.47	23.43	23.45	0.03	2	-1	
24.78	24.63	24.70	0.11	4	-2	
25.29	25.51	25.40	0.15	8	-3	
26.50	26.30	26.40	0.14	16	-4	
pta						
21.95	21.98	21.97	0.02	1	0	97.56
22.82	22.98	22.90	0.11	2	-1	
23.66	24.00	23.83	0.24	4	-2	
25.04	25.35	25.19	0.22	8	-3	
25.95	26.06	26.00	0.08	16	-4	
prpB						
22.29	22.31	22.30	0.01	1	0	100.32
23.60	23.26	23.43	0.24	2	-1	
24.84	24.27	24.55	0.40	4	-2	
25.37	25.34	25.35	0.02	8	-3	
26.18	26.46	26.32	0.19	16	-4	
prpC						
20.01	20.04	20.02	0.02	1	0	103.60
20.94	20.77	20.86	0.12	2	-1	
22.01	21.76	21.88	0.18	4	-2	
22.90	23.02	22.96	0.09	8	-3	
23.57	23.88	23.73	0.22	16	-4	
prpD						
19.88	19.91	19.89	0.02	1	0	98.03
21.18	20.79	20.98	0.27	2	-1	
22.02	21.73	21.88	0.21	4	-2	
22.98	22.81	22.90	0.12	8	-3	
24.05	24.12	24.09	0.04	16	-4	
prpE						
23.04	22.77	22.90	0.19	1	0	98.13
24.03	23.58	23.80	0.32	2	-1	
24.82	25.05	24.93	0.16	4	-2	
25.74	26.02	25.88	0.20	8	-3	
26.81	27.20	27.01	0.28	16	-4	

prpR						
22.32	22.29	22.30	0.02	1	0	104.37
23.21	23.05	23.13	0.11	2	-1	
23.91	24.16	24.03	0.17	4	-2	
25.14	25.21	25.18	0.05	8	-3	
25.99	25.99	25.99	0.01	16	-4	
gyrA (reference gene)						
20.96	20.88	20.92	0.03	1	0	96.76
22.18	21.74	21.96	0.16	2	-1	
22.99	22.73	22.86	0.09	4	-2	
24.16	23.85	24.01	0.11	8	-3	
25.23	25.06	25.14	0.06	16	-4	

SD – standard deviation

PCR primer efficiency was calculated as

Efficiency (%) = $(2^{-1/\text{slope value}}-1)*100$

This data was used to plot standard curves and calculate qRT-PCR efficiency for each primer pairs

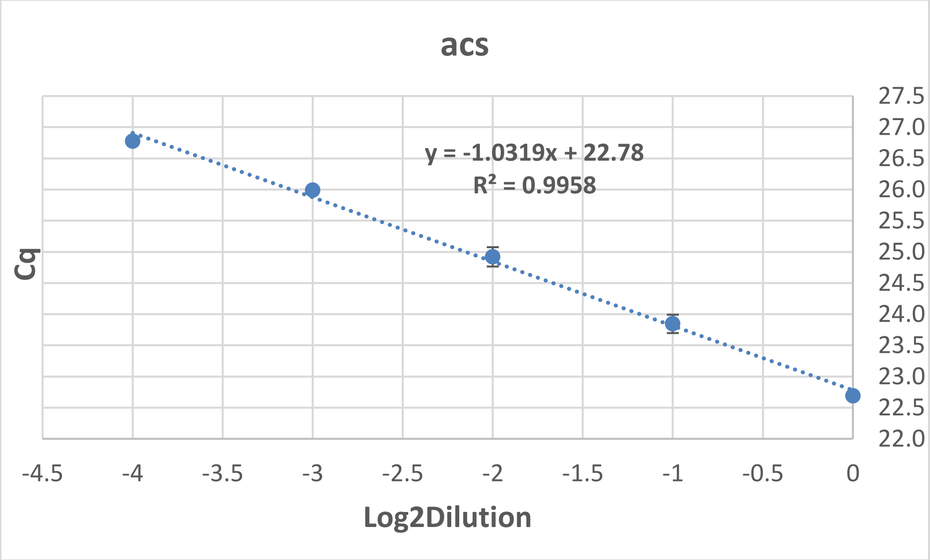

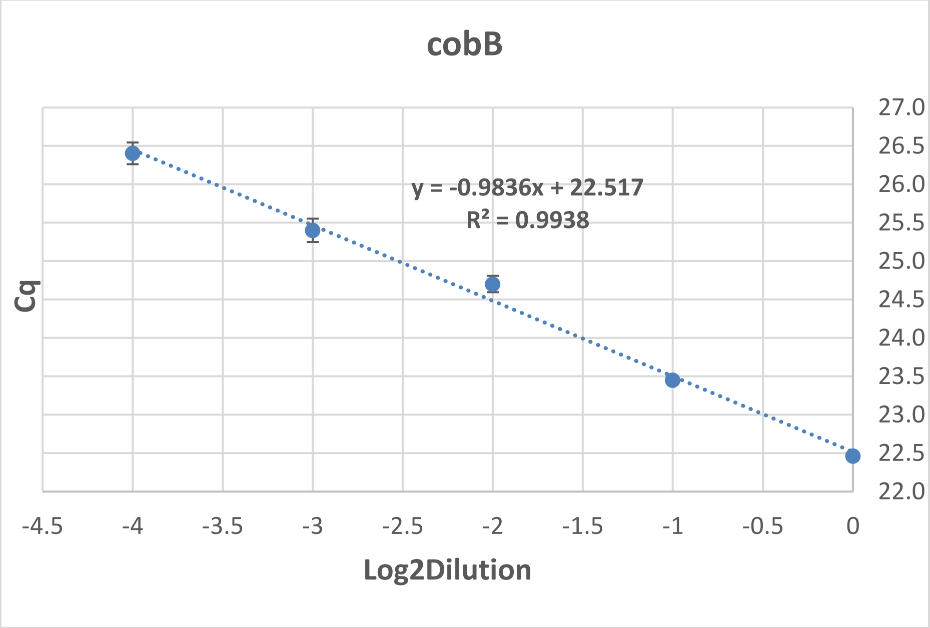
cobB
y = -0.9836x + 22.517
R² = 0.9938
Cq
Log2Dilution
27.0
26.5
26.0
25.5
25.0
24.5
24.0
23.5
23.0
22.5
22.0
-4.5
-4
-3.5
-3
-2.5
-2
-1.5
-1
-0.5
0

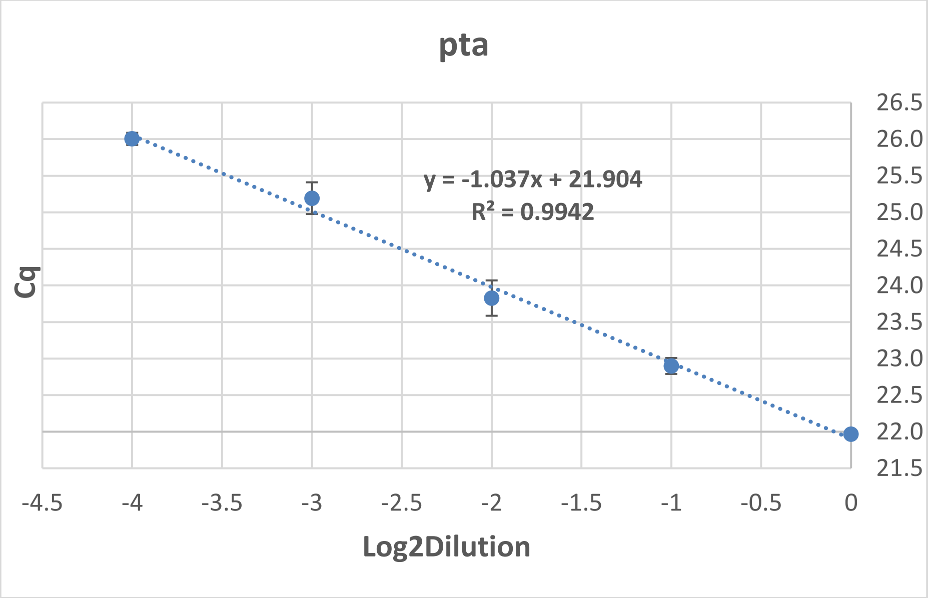
pta
y = -1.037x + 21.904
R² = 0.9942
Cq
Log2Dilution
26.5
26.0
25.5
25.0
24.5
24.0
23.5
23.0
22.5
22.0
21.5
-4.5
-4
-3.5
-3
-2.5
-2
-1.5
-1
-0.5
0

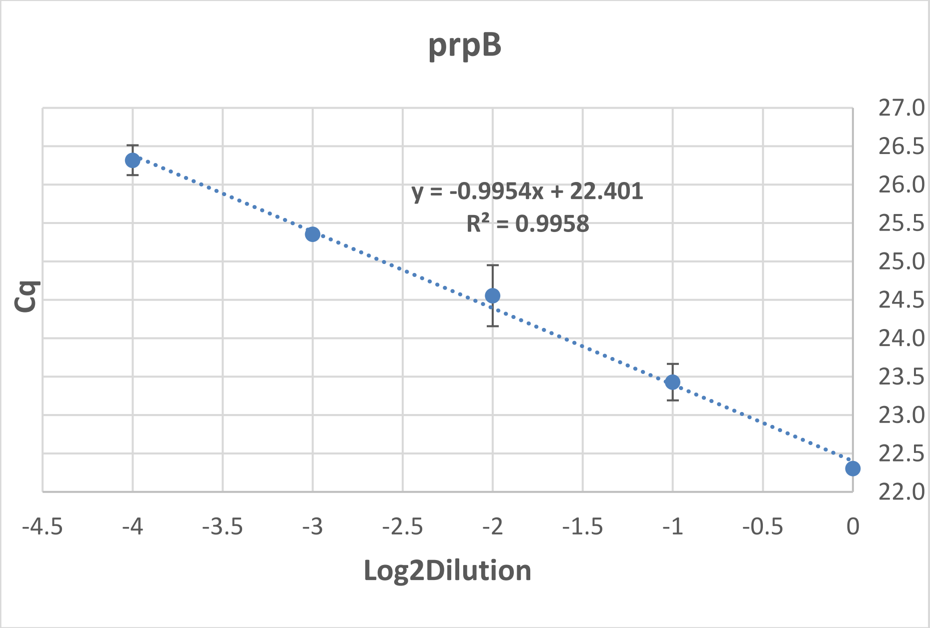
prpB
y = -0.9954x + 22.401
R² = 0.9958
Cq
Log2Dilution
27.0
26.5
26.0
25.5
25.0
24.5
24.0
23.5
23.0
22.5
22.0
-4.5
-4
-3.5
-3
-2.5
-2
-1.5
-1
-0.5
0

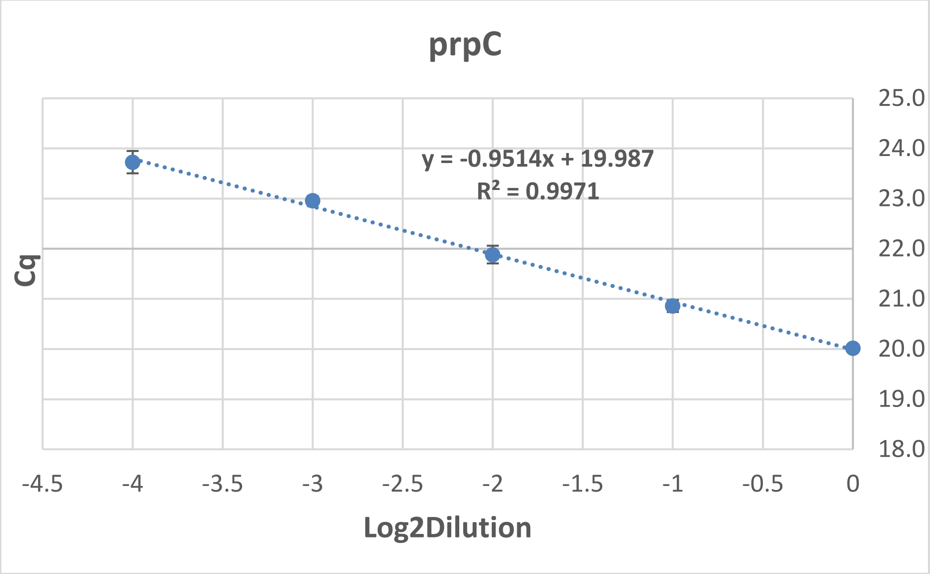
prpC
y = -0.9514x + 19.987
R² = 0.9971
Cq
Log2Dilution
25.0
24.0
23.0
22.0
21.0
20.0
19.0
18.0
-4.5
-4
-3.5
-3
-2.5
-2
-1.5
-1
-0.5
0

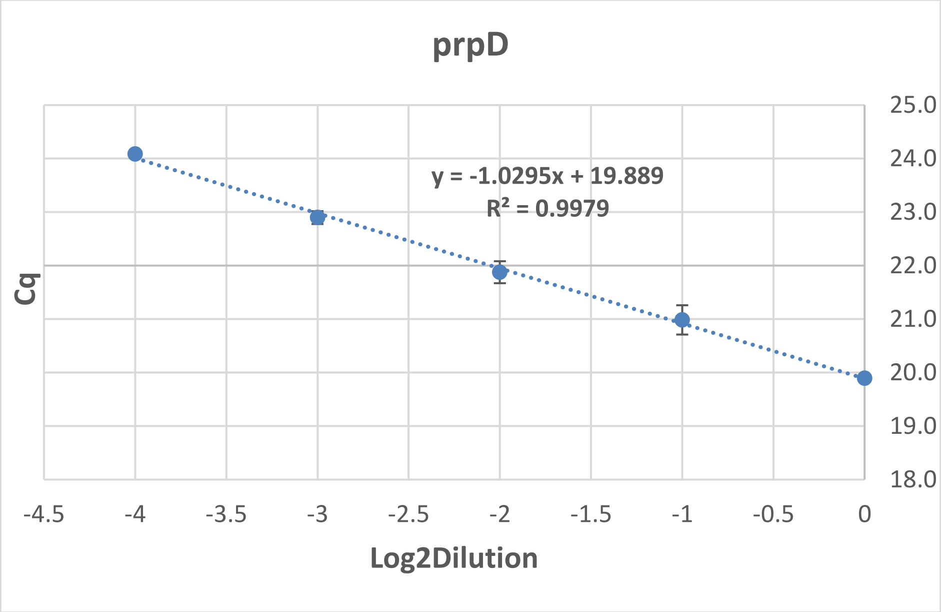
prpD
y = -1.0295x + 19.889
R² = 0.9979
Cq
Log2Dilution
25.0
24.0
23.0
22.0
21.0
20.0
19.0
18.0
-4.5
-4
-3.5
-3
-2.5
-2
-1.5
-1
-0.5
0

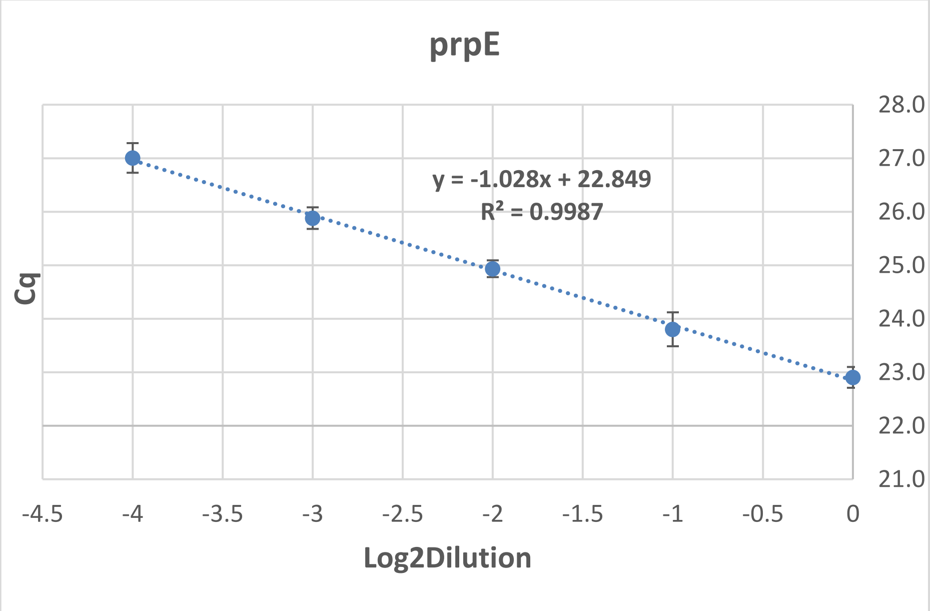
prpE
y = -1.028x + 22.849
R² = 0.9987
Cq
Log2Dilution
28.0
27.0
26.0
25.0
24.0
23.0
22.0
21.0
-4.5
-4
-3.5
-3
-2.5
-2
-1.5
-1
-0.5
0

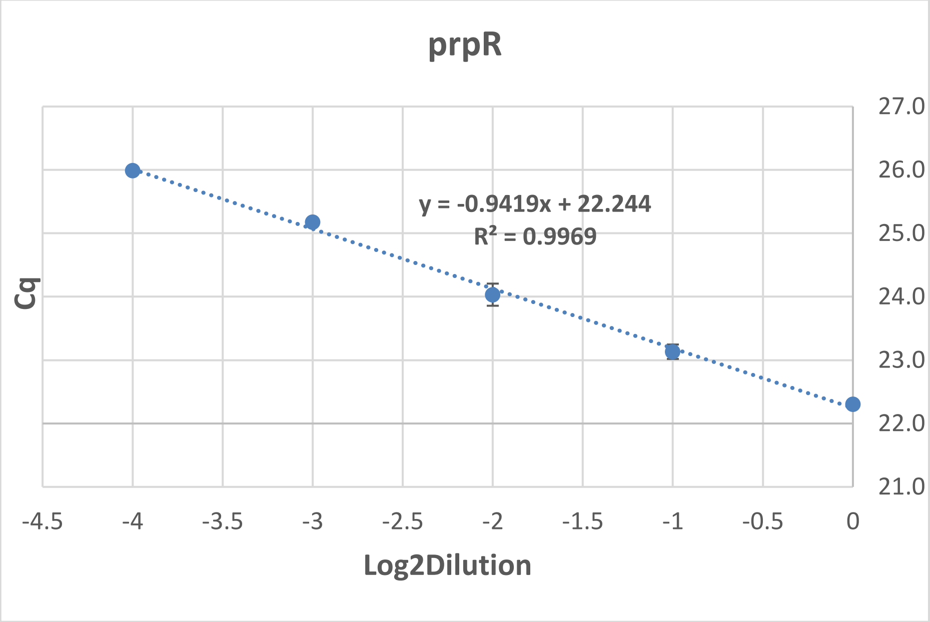

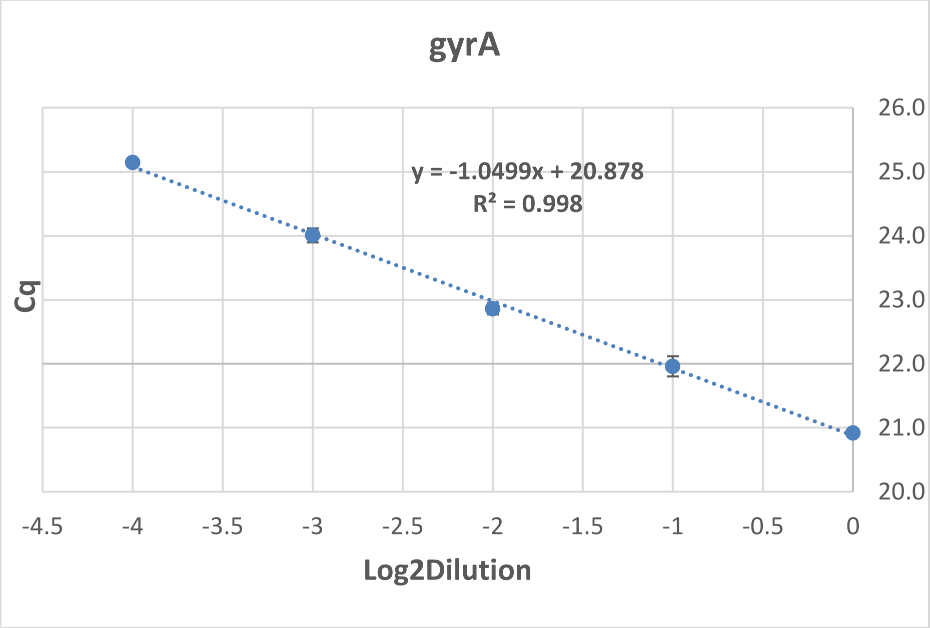

Figure S1 Standard curves for qRT-PCR efficiency

We used R package Growthcurver (Sprouffske K, Wagner A. Growthcurver: an R package for obtaining interpretable metrics from microbial growth curves. BMC Bioinformatics 17, 172 (2016).) for fitting growth curve data by the standard logistic equation common in ecology and evolution (Crow JF, Kimura M. An Introduction to Population Genetics Theory. Caldwell, NJ: Harper and Row, Publishers, Inc.; 1970), (Rockwood LL. Introduction to Population Ecology, 2nd ed. West Sussex: Wiley; 2015). (Figure S1).

The logistic equation gives the number of cells N_t at time t:

$$N_t = \frac{k}{1 + (\frac{k - n_0}{n_0})e^{-rt}}$$

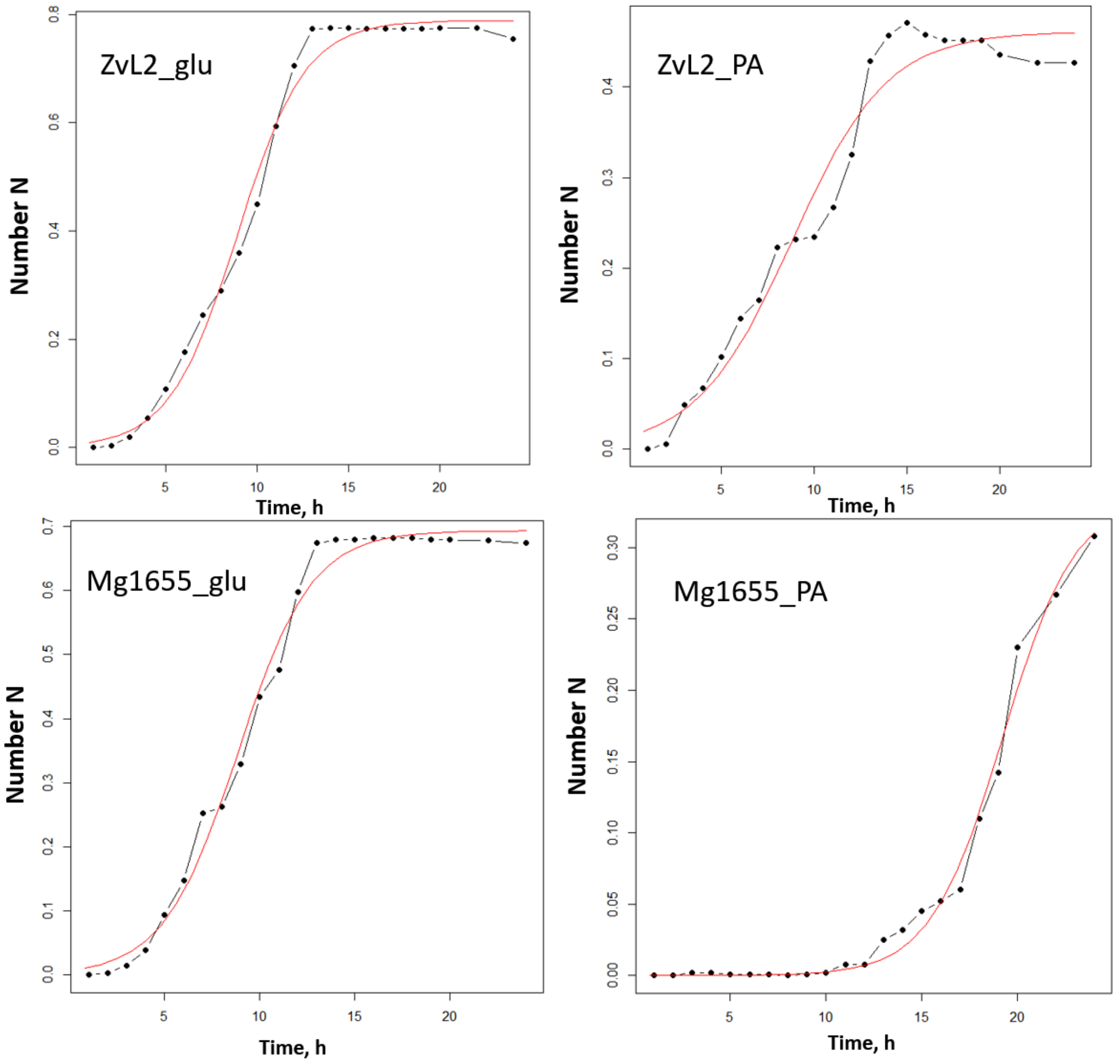

Figure S2. The plot of the raw growth curve data and the best fit logistic curve for ZvL2 isolate (ZvL2) and laboratory strain *E.coli* K12 MG1655 (Mg1655) cultivated on M9 minimal medium with the addition of glucose (Glu) or sodium propionate (PA).

We extracted the value of arguments for all fitting curves (Table S2)

Table S3. The value of arguments

	Samples			
**Arguments**	**ZvL2 glu**	**ZvL2 PA**	**Mg1655 glu**	**Mg1655 PA**
k	0.789	0.461	0.693	0.335
k_se	0.0117	0.0147	0.0103	0.0162
k_p	4.42E-24	7.54E-18	4.19E-24	1.85E-14
n0	0.00632	0.01470	0.00717	0.00001
n0_se	0.0021	0.00556	0.00218	1.03E-05
n0_p	0.00733	0.0163	0.00383	0.251
r	0.541	0.389	0.516	0.531
r_se	0.0383	0.0465	0.0354	0.0475
r_p	1.59E-11	8.62E-08	8.99E-12	8.48E-10
sigma	0.0308	0.0321	0.0265	0.0102
df	19	19	19	19
t_mid	8.9	8.79	8.83	19.2
t_gen	1.28	1.78	1.34	1.3
auc_l	11.9	6.98	10.5	1.65
auc_e	11.9	6.92	10.5	1.68
note				

Description of the arguments

k The carrying capacity parameter
k_se The standard error of the carrying capacity parameter
k_p The p value of the carrying capacity parameter
n0 The initial population size
n0_se The standard error of the initial population size
n0_p The p value of the initial population size
r The growth rate
r_se The standard error of the growth rate
r_p The p value of the growthrate
sigma Residual standard error from non-linear least squares fit of the model to the data
df Degrees of freedom
t_mid The time at the inflection point of the logistic curve (occurs at half of the carrying capacity)
t_gen The maximum doubling time, obtained by evaluating the the unrestrained growth of the population with growth rate r
auc_l The area under the curve of the fitted logistic equation from time 0 to time t
auc_e The area under the curve of the measurements.
note Feedback on common problems with fitting the logistic curve to the data

Table S4. Adjusted P-values for pairwise comparison of all groups in each experiment

Caco2. adherence						
	Mg1655 Glu	Mg1655 initial	Mg1655 PA	ZvL2 Glu	ZvL2 initial	ZvL2 PA
Mg1655 initial	1.59E-02	-	-	-	-	-
Mg1655 PA	6.15E-03	9.10E-04	-	-	-	-
ZvL2 Glu	2.65E-01	9.45E-01	5.03E-02	-	-	-
ZvL2 initial	1.56E-02	6.25E-02	6.15E-03	1.04E-01	-	-
ZvL2 PA	7.30E-04	9.10E-04	7.30E-04	2.04E-03	2.65E-02	-
ZvL2 PA-Glu	5.46E-01	6.15E-03	2.69E-03	2.02E-01	1.42E-02	7.30E-04

Caco2. invasion						
	Mg1655 Glu	Mg1655 initial	Mg1655 PA	ZvL2 Glu	ZvL2 initial	ZvL2 PA
Mg1655 initial	9.97E-03	-	-	-	-	-
Mg1655 PA	7.99E-02	6.60E-04	-	-	-	-
ZvL2 Glu	3.20E-04	4.30E-04	3.50E-04	-	-	-
ZvL2 initial	1.80E-04	1.80E-04	1.80E-04	3.50E-04	-	-
ZvL2 PA	3.50E-04	3.70E-04	3.50E-04	6.30E-04	3.15E-03	-
ZvL2 PA-Glu	2.31E-01	3.04E-01	3.27E-02	3.20E-04	1.80E-04	3.50E-04

Macrophages. 6h postinfection						
	Mg1655 Glu	Mg1655 initial	Mg1655 PA	ZvL2 Glu	ZvL2 initial	ZvL2 PA-Glu
Mg1655 initial	1.60E-05	-	-	-	-	-
Mg1655 PA	5.77E-01	3.80E-06	-	-	-	-
ZvL2 Glu	1.40E-12	6.51E-01	3.80E-09	-	-	-
ZvL2 initial	1.60E-09	2.10E-04	2.20E-11	1.10E-06	-	-
ZvL2 PA-Glu	1.37E-01	7.10E-05	2.38E-01	1.10E-04	1.60E-07	-
ZvL2 PA	7.10E-11	1.00E-06	1.40E-12	8.70E-09	8.80E-04	3.20E-09

Macrophages. 24h postinfection						
	Mg1655 Glu	Mg1655 initial	Mg1655 PA	ZvL2 Glu	ZvL2 initial	ZvL2 PA
Mg1655 initial	1.20E-06	-	-	-	-	-
Mg1655 PA	6.84E-01	7.60E-07	-	-	-	-
ZvL2 Glu	8.40E-08	7.08E-01	6.50E-08	-	-	-
ZvL2 initial	4.20E-07	8.20E-06	3.50E-07	1.00E-05	-	-
ZvL2 PA	3.40E-15	1.40E-12	2.00E-16	7.80E-14	2.90E-02	-
ZvL2 PA-Glu	5.79E-01	7.50E-07	7.37E-01	1.00E-07	1.70E-07	2.00E-16
